# Evaluation of the psychometric properties of *Hindi*-translated Scale for Measuring Maternal Satisfaction among postnatal women in Chhattisgarh, India

**DOI:** 10.1371/journal.pone.0211364

**Published:** 2019-01-29

**Authors:** Paridhi Jha, Margareta Larsson, Kyllike Christensson, Agneta Skoog Svanberg

**Affiliations:** 1 Department of Women's and Children's Health, Uppsala University, Uppsala, Uppsala, Sweden; 2 Department of Women's and Children's Health, Karolinska Institutet, Stockholm, Sweden; Universiteit Gent, BELGIUM

## Abstract

Satisfaction with childbirth services is a multi-dimensional phenomenon, providing relevant insights into women's opinion on quality of services received. Research studies report a dearth of standardised scales that quantify this phenomenon; and none have been tested in India to the best of authors' knowledge. The current study was undertaken to evaluate psychometric properties of *Hindi* version of the Turkish Scale for Measuring Maternal Satisfaction: Normal and Caesarean Births versions in order to fill this gap. A cross-sectional survey was conducted in selected public health facilities in Chhattisgarh, India. Healthy women (n = 1004) who gave birth to a single, live neonate, vaginally or via Caesarean section participated. Psychometric assessment was carried out in four steps: 1) scales translated from Turkish to *Hindi*; 2) Content Validity Index scores calculated for *Hindi* scales; 3) data collection; 4) statistical analyses for *Hindi* scales (Normal and Caesarean Birth).

A 10-factor model with 36 items emerged for both scales. The *Hindi-* translated Normal Birth and Caesarean Birth scales had good internal reliability (Cronbach’s α coefficients of 0.85 and 0.80, respectively).

The *Hindi* Scales for Measuring Maternal Satisfaction (Normal and Caesarean Birth) are valid and reliable tools for utilization in Indian health facilities. Their multi-dimensional nature presents an opportunity for the care providers and health administrators to incorporate women's opinions in intervention to improve quality of childbirth services. Having an international tool validated within India also provides a platform for comparing cross-country findings.

## Introduction

Institutional childbirth has been promoted globally as a means of eliminating preventable causes of maternal and newborn deaths [1]. The quality of institutional childbirth-related services has been known to influence the women's and their families' choice of birthplace [2–5]. Some components of quality–the infrastructure of the delivery room, supply of articles, provider-to-woman ratio, etc.–are tangible and accurately measureable. However, components such as the behaviour of care providers, comfort and how easily the services can be obtained by women inside the labour rooms, and overall support provided by health workers during childbirth are subjective in nature, and are difficult to measure without analyzing the women’s own accounts of services received.

Likert scales are commonly used tools to measure people’s opinions and experiences in health sciences [6]. In relation to childbirth, Likert scales are often used to accurately quantify women’s experiences and opinions related to services received. This knowledge guides the care providers in caring for women during institutional births in a socio-culturally appropriate manner. It may also reveal malpractices, and assist in improving women's satisfaction with institutional childbirth. Several studies from high, Middle, and low income countries show that satisfaction with childbirth and service received is adversely associated with having fear of childbirth and/or postnatal depressive symptoms [7–9]; at the same time, women who are less satisfied with childbirth and services received, more commonly face difficulty with establishing mother-baby bond, initiating breastfeeding and are less likely to comply with self and baby care advice compared to women who are satisfied with their childbirth and services they received [10–12]

With increasing attention towards client satisfaction as a key indicator of quality of services [13,14]; measuring client satisfaction–including those of women giving birth–has gained global momentum [15]. To this end, the Government of India has also implemented several reforms to make the childbirth services more women-friendly [16,17]. However, there exists the need of a standard tool to assess Indian women’s satisfaction with childbirth services. This is important, as the Government of India has strived for, and succeeded in, attracting ever-increasing numbers of women to health facilities for childbirth. The few studies available on satisfaction with health care and/or childbirth services are qualitative in nature [5,18,19], or have used a locally-designed quantitative tool without adequate psychometric testing. To the best of our knowledge, no standard scale has been developed, or tested, in India to measure Indian women’s satisfaction with childbirth services.

Review of literature from other countries shows that several scales have been developed across the world to measure women’s satisfaction with childbirth services:

### Mackey childbirth satisfaction rating scale

This scale, developed by M. Mackey and P. Goodman, reports the multi-dimensional nature of childbirth satisfaction [20]. The 40-item, 5-point Likert scale has six sub-dimensions: general satisfaction, satisfaction with self, baby, midwife, physician and partner. The original scale in English has been widely translated and psychometrically validated [21–24] to measure childbirth satisfaction for both normal and caesarean births (internal reliability range 0.76–0.84 in all translations).

### Quality from the patients’ perspective (QPP-I)

This theory-based satisfaction questionnaire has its roots in patients’ perceptions. The tool was developed by B. Wilde-Larsson et al. in 1994 [25], and was subsequently refined and simplified [26–28]. The multi-dimensional QPP (short form) has 24 items under four dimensions: medical-technical competence, physical-technical conditions, identity-oriented approach and socio-cultural aspects (internal reliability range 0.73–0.93 in long and short forms).

### The birth satisfaction scale

The BSS was developed by C. Hollins Martin and V. Fleming in 2009 after a systematic review of research-based childbirth satisfaction and dissatisfaction expressions [29]. This 30-item, 5-point Likert scale has three over-arching themes and 15 sub-themes.

### Newcastle satisfaction with nursing scale (NSNS)

This scale was developed by L. Thomas et al. in 1996 to measure how women experienced the nursing care provided to them [30]. The 5-point Likert scale has two subscales: experience with nursing care and satisfaction with nursing care. The 19-item sub-scale measuring patient satisfaction has good internal reliability (0.96).

### Satisfaction with maternal and newborn health care following childbirth

This 11-item scale was developed by F. T. Camacho et al. in 2012 to assess women’s satisfaction with health care services received in the weeks following childbirth [31]. This single-factor scale has a high internal validity score (0.96). The authors disclose that this scale is the first validated scale to measure the satisfaction of women for both self-care and care of their newborn baby following the childbirth.

### Scale for Measuring Maternal Satisfaction (SMMS)

The SMMS has two versions [32]; Normal Birth (content validity index score 0.91), and Caesarean Birth (content validity index score 0.89). The scale-version Normal Birth has 43 items, while the scale-version Caesarean Births has 42 items, rates over a 5-point Likert scale. Cut-off scores for Normal Birth and Caesarean Birth are 150.5 and 146.5, respectively, where scores above the cut-off indicate greater satisfaction. The scales (both Normal and Caesarean Birth) have 10 sub-scales each: i) Perception of health professionals; ii) Nursing care in labour; iii) Comforting; iv) Information and involvement in decision making; v) Meeting baby; vi) Postpartum care; vii) Hospital room; viii) Hospital facilities; ix) Respect for privacy and x) Meeting expectations. The Turkish SMMS Normal and Caesarean Birth scales have 13 and 12 items respectively that need to be reverse-coded before calculating final scores.

### The childbirth context in indian public health facilities

The National Family Health Survey 4 report (2015–2016) shows that approximately 67% women in reproductive age group are literate; and the average age at first pregnancy among women is 20.1 years [33]. The institutional childbirths in India have doubled from 39% in 2005–06 to 79% in 2015–2016 [33]. Out of all live births in 2009–2013, 17% were by Caesarean section. Public health facilities alone are estimated to cater to two-thirds of all childbirth service-needs [33]. Indian public health delivery system has following levels of health facilities [34]: Medical College hospitals (> 500 bedded; childbirths/year: ≥ 10,000); District Hospitals (100–500 bedded; childbirths/year: could go as high as 10,000 or more); Community Health Centers (30–60 bedded facilities, Childbirths/year: very varied); Primary Health Centres (6–10 bedded facilities, not every facility provides childbirth services). The labour rooms in the Medical College Hospital; District Hospitals and Community Health Centres are often built as long halls with several birth cots arranged in the Nightingale ward layout; with curtains or detachable privacy screens built around the cot to provide privacy. Most commonly, the toilets are towards one end of the hall and the nurse-midwives' work station; and equipment are organized at the other end of the hall. Labour rooms at Primary Health Centres have one or two cots; and a woman may be the only occupant while in labour; as childbirth loads are significantly lower compared to Community Health Centres and higher facilities. Approximately 81% of all pregnant women have access to skilled birth attendance, irrespective of place of birth [33]. Physicians are the most common service providers during childbirths (52%) followed by nurse-midwives and Auxiliary Nurses and Midwives (25%) [33]. Pain relief is not offered to women in labour; and evidence from different part of India suggests that Indian women prefer experiencing pain; associating it to faster childbirth [35–38].

### Selection of tool for this study

Keeping this context in mind, The Mackey Childbirth Satisfaction Rating Scale; and The QPP-I were both found not suitable as they had questions pertaining to availability of private rooms and toilets and other facilities more commonly associated with individual birthing rooms.

The Newcastle Satisfaction with Nursing Scale was suitable for the generic opinion of care, but did not elicit responses unique to childbirth (labour and immediate postnatal period). The authors of Birth Satisfaction Scale acknowledged the possibility of ambiguity in responses upon using the questionnaire in its published form [29]; and Satisfaction with maternal and newborn care following childbirth recorded women's opinion from the post-natal period; with no responses targeting labour or actual birth.

Out of all scales explored, Scale for Measuring Maternal Satisfaction came closest to capture the labour and birth scenario from Indian context. Also, posing SMMS alongside QPP and Mackey during a pre-study face validity exercise among a representative group of women showed that women found SMMS easier to comprehend. Psychometric properties of the original tool have been reported by the authors [32]; and sub-scales identified were also appropriate in an Indian context.

Keeping these pre-research assessments in mind, SMMS was selected for translation and use in this study, with an aim to evaluate the psychometric properties of the *Hindi-*translated SMMS Normal and Caesarean Birth scales. The null hypothesis was that the construct validity of *Hindi-*translated SMMS Normal and Caesarean Birth Scales will be same as the original Turkish versions.

### Socio-cultural adaptation of SMMS to suit Indian context

Few changes were made in terminology used in the questionnaire after email consultation with original authors: 1) Term 'partner' was replaced with 'husband'; (no unmarried woman gave birth at selected facility in past 5 years as per hospital census); 2) presence of husband near woman during her hospital stay for labour and childbirth was replaced with presence of family (family members–mostly married females–accompany woman inside labour room/postnatal wards. Husbands are not allowed entry to the labour room). All other services were described in the original tool as they are also provided in India; thus negating the need for significant changes in original tool.

## Material and methods

### Sample and setting

A cross-sectional survey in public health facilities of two districts of the Chhattisgarh State, India was carried out to achieve the objective. The STARD guidelines were followed to report findings.

All women, admitted to the postnatal wards of selected public health facilities were eligible to participate. The exclusion criteria were, women who: 1) had had a stillbirth, and/or 2) multiple pregnancy and/or; 3) had a prolonged complicated childbirth followed by the physicians’ orders to not to be disturbed. All other women who had given birth at the selected health facility, and were not under any observation lists of physicians except the routine medical rounds were considered healthy; and invited to participate. Data collection was carried out at two levels of health facilities in Chhattisgarh state: 1) District Hospitals (DHs), which offered services for vaginal as well as caesarean births and had approximately 20–25 childbirths per day during the study period, and 2) the Community Health Centres (CHCs), offering care to women having vaginal childbirths, with 10–200 institutional births per month per facility. None of these CHCs offered caesarean section services during the data collection period. The two DHs–only one per district is available–from two districts were selected. Out of 22 total CHCs from both districts, 17 CHCs having 10 or more institutional childbirths per month were selected. Non-probability consecutive sampling was used to recruit participants.

### Sample size

**Fig 1:**
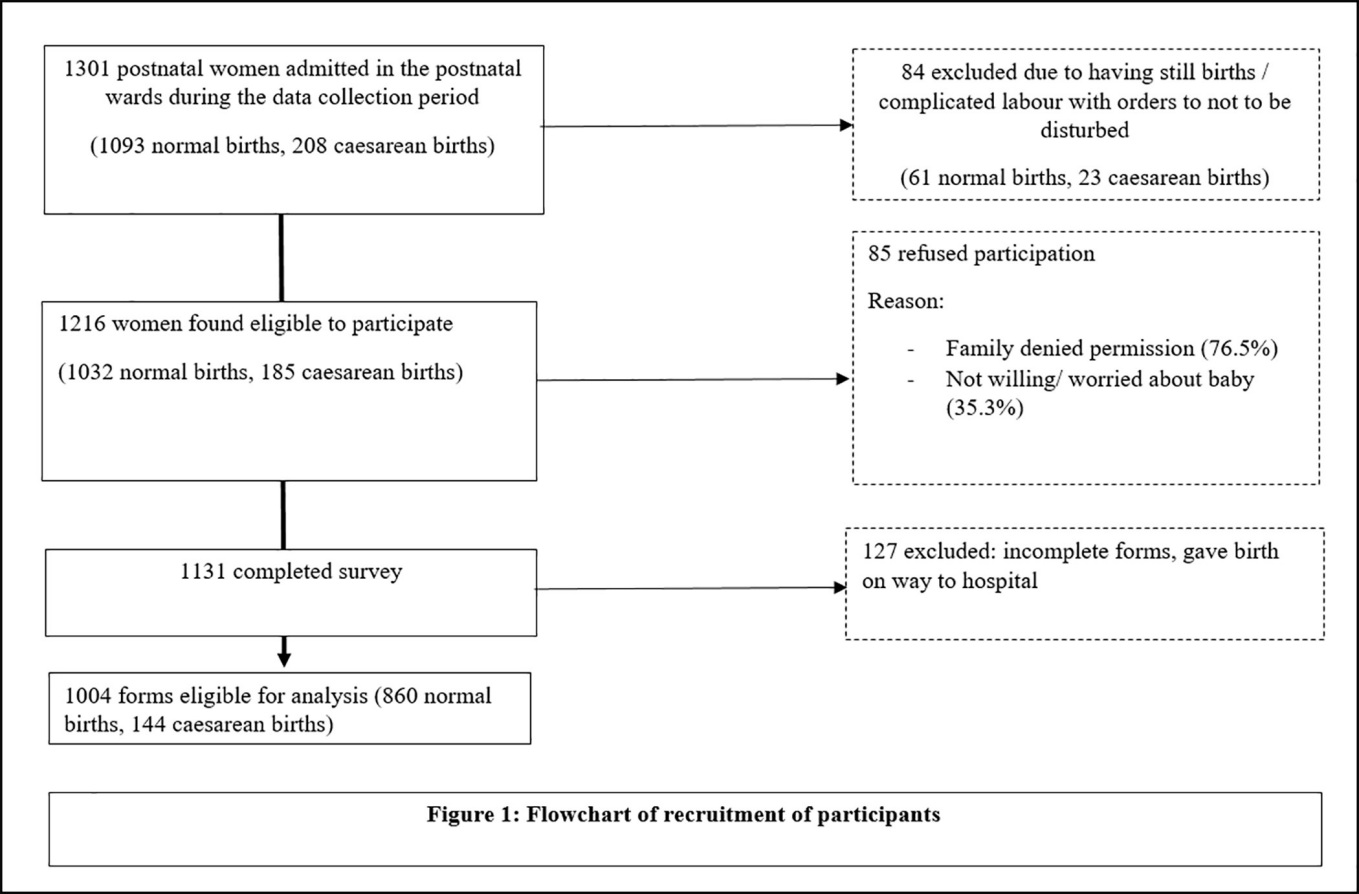
Flowchart of Recruitment of Participants.

A pilot study was carried out among 100 participants at one district hospital and one CHC (not selected for actual study) by the first author; with 70% response rate of completing interviews. Review of literature suggested, a sample size of 500 is considered good, whereas a sample size of 1000 is considered exceptionally good for psychometric evaluation [6]. Adjusting for 30% dropouts experienced in pilot study, 1216 participants were recruited for data collection and 1131 completed data collection. After discarding non-completed questionnaires; questionnaires that had missed item rates of more than 3% or questionnaires with more than 2 items missed by a participant; and the questionnaires of those who had actually given birth on the way to hospital, a total of 1004 completed questionnaires remained (Vaginal birth *n* = 860 and Caesarean births *n* = 144). Diagram 1 depicts the flow of participants' recruitment (Fig 1). Data collection commenced in March 2015 and continued until May 2015.

### Scale validation process

#### Step 1: Ensuring the understandability of the scales among Hindi-speaking women

The scales were translated from Turkish to *Hindi* by a professional translator and were linguistically validated through reverse-translation by a separate linguistic expert. Cognitive interviews (using the read-aloud method) were carried out with 25 postpartum women to test the face validity of the *Hindi-*translated scales; and, to determine the understandability of the questionnaires by the respondents. Women voiced everything that came to their mind after each question was read aloud to them and the first author recorded all new perspectives on each item. The level of comprehension was similar between illiterate and literate women during the cognitive interviews. Though the literate women could answer the questionnaire on their own; many preferred responding to an interviewer. To keep the data collection technique similar across participants, one-to-one interviews were selected as the most acceptable method to collect data.

#### Step 2: Calculating content validity index scores

The scale was presented to one obstetrician, one gender and rights expert, and four midwifery experts (postgraduate degree in Gynecological Nursing and Midwifery, or in Public Health), who rated each item on a scale of 1–3 (1 being ‘not relevant’; 2, ‘somewhat relevant’; and 3, ‘relevant’). CVI calculations advise retaining items having CVI score of 0.8 or above, modifying items having CVI 0.70 to 0.80; and deleting items having CVI index of less than 0.70 [6]. Based on ratings, a content validity score was obtained for each item on the scales. The minimum acceptable Content Validity Index was set at 0.80 (at least 80% of reviewers' agreement upon an item's relevance): Ratings of 2 or 3 were considered to be relevant [39]. Items with CVI <0.70 were deleted.

#### Step 3: Ethics and consent processes

Consent to translate the scales in *Hindi* for use in India was sought from the authors of the original scale. Ethical clearance and approval for data collection was sought from the State Government of Chhattisgarh. The Patient Welfare Committee at each selected facility approved the schedule for data collection so that the service delivery and 'visiting-time' for families were not interrupted. Participants received detailed written information/information was read out to participants in *Hindi* about the research study, and all signed an informed consent form. Data were collected through one-to-one interviews so that illiterate women had equal chance to participate. Anonymity of the participants was ensured at all times. Participants were aware that there would be no monetary incentive for participating in the study, and that they could refuse to participate or stop participation at any time.

#### Step 4: Data collection

Ten female research assistants (RAs)–aged between 22–34 years–having a bachelor's degree and having previous training and experience of data collection in large-scale National Family Health Survey (Government of India) were interviewed and recruited. All respondents could speak *Hindi*. One week long training was carried out to train the RAs on study context, objectives, methods of data collection, orientation to questionnaire, classroom based mock interviews and field visits for test-interviews under observation of first author. Seven RAs were retained after the training. Data collection was carried out between March-May 2015 for the current study.

#### Step 5: Psychometric evaluation of Hindi-translated questionnaires

All analyses were carried out on IBM software SPSS 24. The following processes were performed for the psychometric evaluation:

Item analysis: item analysis was carried out for both scales to calculate the strength of relationship between an item and its relevance for a satisfaction scale. The acceptable range of the correlation coefficient was set between >0.25 and <0.75 [6]. Items having a correlation coefficient of less than 0.25 were deleted from *Hindi-*scales [32].

Construct validity: exploratory principal component factor analysis (varimax rotation) was carried out to check the construct validity of both translated versions of SMMS to reduce the number of ambiguous or non-contributing items from a scale [40]; and, to identify factors or dimensions that each explain one aspect of the satisfaction scale [6,39]. Sample adequacy for both scales was confirmed through the Kaiser-Meyer-Olkin test. All emerging factors having an eigenvalue >1 were explored to develop a component matrix. Item coefficients were limited to ≥ 0.4 to avoid multi-factor associations. Items with multiple loading were deleted, thus each factor identified in the exploratory principal component analysis represented only one sub-scale or dimension of childbirth satisfaction.

Internal reliability: Cronbach’s α coefficient calculates the degree to which a set of items are interrelated for measuring a single construct [39]. An internal reliability score of > 0.8 is expected for a well-constructed scale, and a score of > 0.7 is considered fair for a newly constructed scale. Each emerging sub-scale extracted from the factor analysis was evaluated to check the correlation with the total scale scores. Correlation scores of a subscale were also calculated with each item that fell into that subscale.

Divergent validity: divergent validity establishes whether two constructs known to have no relationship, in fact, do not have a statistical relationship. [6] A validated scale measuring another concept is administered alongside the scale being tested. Low correlations between the total scores of the two scales show that the scale being tested, is, in fact, testing a different concept than the other scale. For this study, Edinburgh Postnatal Depression Scale (EPDS), which has previously been translated and validated in India [41], was used to perform the divergent validity check.

Cut-off scores: Cut-off scores were determined using ROC curve analysis; where score of 3.5 and above indicated higher level of satisfaction and score below 3.5 indicated lower level of satisfaction with services.

## Results

Each interview took, on average, 18 minutes to complete. The response rate was 93% with most refusals being due to family members’ unwillingness to let the women participate.

### Participants' characteristics

The mean (standard deviation) ages of women participating in this study were 23.64 years (SD 3.51) and 23.98 years (SD 3.35) for vaginal and Caesarean births, respectively. A very small proportion (7.8%) of participants could not read or write in any language. Women gave birth vaginally or through Caesarean birth. Out of all who had vaginal birth, approximately one-third received an episiotomy/experienced a tear requiring suturing. No vacuum or forceps extraction was performed for any respondent. During this study, no clear records were found on augmentation of labour, indication, medicine used; its quantity or method of administration. Table 1 presents the demographic characteristics and obstetric history of the participants.

**Table 1:** Demographic characteristics and obstetric history of the participants.

	Vaginal birth*	Caesarean birth*
Mean Age in years (std. deviation)	23.64 (3.51)	23.98 (3.35)
Mean age at marriage (std. deviation)	19.43 (2.13)	20.19 (3.07)
Education (n = 859 vaginal; n = 138 CS)		
Never been to school (Illiterate)	71 (8.3)	8 (5.7)
Up to 8 years' formal education	415 (48.3)	66 (47.1)
Up to 12 years of formal education	328 (38.1)	47 (33.6)
Attending/ attended college	45 (5.2)	17.8 (12.1)
Working status (n = 856 vaginal; n = 139 CS)		
Homemaker	612 (71.2)	79 (56.4)
Earning own salary	244 (28.4)	61 (43.6)
Social category (n = 851 vaginal; n = 138 CS)		
Scheduled caste	118 (13.7)	28 (20.0)
Scheduled tribe	144 (16.7)	12 (8.6)
Other Backward Classes	496 (57.7)	88 (62.9)
General	93 (10.8)	8 (5.7)
Perception about self-health (n = 850 vaginal; n = 139 CS)		
Good	742 (86.3)	121 (86.4)
Not good	108 (12.6)	18 (12.9)
Gravidity (n = 860 vaginal; n = 140 CS)		
Primi	382 (44.4)	66 (47.1)
Multi	478 (55.6)	74 (52.9)
Parity (n = 860 vaginal; n = 140 CS)		
Primi	406 (47.2)	70 (50.0)
Multi	454 (52.8)	70 (50.0)
Experienced spontaneous abortion before this birth (n = 860 vaginal; n = 140 CS)		
Yes	49 (5.7)	6 (4.3)
No	811 (94.3)	134 (95.7)
Lost a child in past (n = 860 vaginal; n = 140 CS)		
Yes	63 (7.3)	15 (10.7)
No	797 (92.7)	125 (89.3)
Gender of the present newborn baby (n = 860 vaginal; n = 140 CS)		
Female	411 (47.8)	72 (51.4)
Male	449 (52.2)	68 (48.6)

*figures in the columns represent number of participants (%) where applicable

### Content validity

In this study, the content validity index (CVI) scores ranged from 0.67 to 1 for the draft forms of the *Hindi-*translated SMMS Normal and Caesarean Birth. The initial total scale CVI for SMMS Normal Births and Caesarean Births was 0.83 and 0.87 respectively. Deleting items with low CVI scores (< 0.80) removed three items from SMMS Normal Birth (‘more things could be done for pain management’; ‘I was informed about all necessary procedures during labour and childbirth’; ‘my family had a comfortable place to stay at hospital’). Another item (‘enough doctors, midwives and nurses were involved in my care’) from SMMS Normal Birth was incomprehensible for the women during the read-aloud sessions, and was removed.

Four items with low CVI scores were also removed from SMMS Caesarean Birth: ‘there were enough doctors, midwives and nurses involved in my care’; ‘doctors did all necessary medical interventions during childbirth’; ‘nurses spent enough time to help breastfeeding’; and ‘there were people coming in and out of my room unnecessarily after the birth’). The participants understood all items during read-aloud sessions. Removing the low CVI items from the scales brought the total scale CVI to 0.90 and 0.93 for SMMS Normal Birth and Caesarean Birth, respectively.

### Item analysis

All remaining items had positive and statistically significant item-total coefficients, which were > 0.25 and < 0.80. Therefore, the item analysis did not result in the deletion of any more items. The scales after item analysis retained 39 and 38 items respectively for Normal Births and Caesarean Births.

### Construct validity

The Kaiser-Meyer-Olkin index, 0.83 and 0.81 respectively for the *Hindi*-translated SMMS Normal and Caesarean Births, showed that the sample size was adequate for factor analysis. Bartlett’s sphericity test results demonstrated that the dataset was appropriate for factor analysis (SMMS Normal Birth: χ^2^ = 6663.31, *p* <0.001; and SMMS Caesarean Birth: χ^2^ = 3807.40, *p* <0.001).

Performing exploratory principal components factor analysis with varimax rotation yielded 10 factors that had eigenvalues of > 1.0 that together explained 70.6% and 71.3% of the variances for *Hindi-*translated SMMS Normal Births and Caesarean Births, respectively. Factor loadings for all items were sufficient (≥ 0.40) in both scales. Three items in SMMS Normal Births showed double loadings: ‘I would have liked more support for stress reduction’; ‘my family could receive more attention for stress reduction’; and ‘we could easily find everything we needed in the hospital’. Similarly, one item in SMMS Caesarean Birth showed double loadings: ‘doctors and nurses explained to me everything about Caesarean birth before operation’. At the same time, in the SMMS Caesarean Birth scale, the item ‘Special moments I lived with my family before and after CS birth were interrupted’ failed to load under any dimension. All double-loaded or no-loading items were deleted. Both questionnaires had 36 items each after factor analysis. Tables 2 and 3 present a summary of the principal component analyses for *Hindi-*translated SMMS Normal Births and SMMS Caesarean Births, respectively. In *Hindi*-translated SMMS Normal Births, items were pooled under 10 dimensions: facilities and services, postpartum care received, information and involvement in decision making, meeting the baby, intrapartum care received, overall support received, meeting expectations from institutional birth, maintaining privacy, compassion and respect during services, and experiences of having an institutional birth.

**Table 2:** Summary of factor analyses for 36- item scale for Measuring Maternal Satisfaction: Normal birth (Hindi translation).

		Number of items	Item analysis			Construct validity (factor analysis)			Internal reliability Cronbach's α
			Item- total correlation Range	Item-subscale correlation Range	Subscale- total correlation Range	Eigenvalue	% explained variance	Loading range	
Factor 1	Hospital facilities	5	0.37–0.50	0.69–0.89	0.52	8.598	19.995	0.52–0.85	0.88
Factor 2	Postpartum Care	6	0.39–0.68	0.50–0.91	0.75	5.807	13.504	0.43–0.86	0.85
Factor 3	Information received & involvement in decision making	5	0.39–0.51	0.76–0.85	0.56	3.868	8.995	0.60–0.84	0.83
Factor 4	Meeting the baby	3	0.43–0.47	0.95–0.97	0.47	2.815	6.548	0.91–0.95	0.95
Factor 5	Intrapartum Care	6	0.39–0.63	0.57–0.79	0.75	2.256	5.246	0.55–0.74	0.75
Factor 6	Overall support received	2	0.30–0.41	0.60–0.76	0.48	1.946	4.526	0.52	0.79
Factor 7	Expectations from institutional birth	3	0.27–0.39	0.66–0.75	0.44	1.573	3.657	0.49–0.76	0.76
Factor 8	Privacy	2	0.26–0.28	0.83–0.89	0.59	1.298	3.019	0.75–0.87	0.72
Factor 9	Compassion and respect	2	0.32–0.33	0.77–0.83	0.54	1.140	2.651	0.45–0.65	0.71
Factor 10	Experiences of having institutional birth	2	0.28–0.29	0.58–0.85	0.69	1.062	2.469	0.40–0.84	0.70
	Total Scale	36				Range 1.06–8.59	70.610	0.40–0.95	0.85

**Table 3:** Summary of factor analyses for 36- item scale for Measuring Maternal Satisfaction: Caesarean birth (Hindi translation): Original work.

		Number of items	Item analysis			Construct validity (factor analysis)			Internal reliability Cronbach's α
			Item- total correlation Range	Item-subscale correlation Range	Subscale- total correlation Range	Eigenvalue	% explained variance	Loading range	
Factor 1	Postpartum Care	4	0.45–0.61	0.76–0.93	0.62	7.428	20.633	0.72–0.89	0.89
Factor 2	Meeting the baby	3	0.96–0.97	0.96–0.97	0.69	3.273	9.090	0.85–0.89	0.96
Factor 3	Information received and involvement in decision making	5	0.26–0.61	0.54–0.84	0.43	2.746	7.627	0.65–0.85	0.63
Factor 4	Overall support received	3	0.36–0.81	0.42–0.61	0.49	2.719	7.553	0.46–0.77	0.66
Factor 5	Stress and discomfort	3	0.36–0.62	0.69–0.89	0.62	2.323	6.453	0.58–0.78	0.74
Factor 6	Intrapartum Care	3	0.78–0.91	0.78–0.90	0.51	1.875	5.209	0.60–0.86	0.78
Factor 7	Hospital facilities	6	0.29–0.51	0.38–0.69	0.48	1.662	4.615	0.47–0.85	0.82
Factor 8	Privacy, compassion and respect	4	0.30–0.46	0.56–0.68	0.67	1.602	4.449	0.46–0.76	0.69
Factor 9	Expectations from institutional birth	3	0.26–0.29	0.62–0.78	0.59	1.271	3.531	0.47–0.83	0.73
Factor 10	Experiences of having institutional birth	2	0.28–0.56	0.37–0.96	0.55	1.137	3.159	0.41–0.74	0.72
	Total Scale	36			Range 0.43–0.69	1.1–7.4	71.3	0.41–0.89	0.80

In *Hindi*-translated SMMS Caesarean Births, items were pooled under 10 dimensions: postpartum care received, meeting the baby, information and involvement in decision making, overall support receive, managing stress and discomfort, intrapartum care received, facilities and services, compassion and respect during services, meeting the expectations from institutional birth, and experiences of having an institutional birth.

### Subscales’ item-analysis

The finalized scales were re-evaluated for item-total, item-subscale and subscale-total correlations. The correlations on both the final *Hindi*-translated scales remained positive, more than 0.25 in value and statistically significant (*p* <0.001). Tables 2 and 3 present the detailed findings.

### Internal reliability

As displayed in Tables 2 and 3, the internal reliability for both *Hindi*-translated final questionnaires was satisfactory (Cronbach’s α coefficients of 0.85 and 0.80 for SMMS Normal and Caesarean Births, respectively). The Cronbach’s α coefficients for subscales ranged from 0.70–0.95 for SMMS Normal Births and 0.63–0.96 for SMMS Caesarean Births.

### Divergent validity

The scales showed poor correlations with the EPDS (SMMS Normal Births *r* = 0.237 and *p* <0.001; SMMS CS Births *r* = 0.142 and *p* <0.001, thus establishing divergent validity.

### Cut-off scores

As in the original scale’s psychometric testing, the cut-off scores were calculated using ROC curve analysis based on the assumption that item scores > 3.5 indicated satisfaction while item score < 3.5 indicated dissatisfaction. According to this hypothesis, the cut-off score for the *Hindi*-translated SMMS Normal Births was calculated as 105.5 (Area under ROC curve 0.79, 95% CI 0.75–0.82, sensitivity 0.985, specificity 0.787); while the cutoff score for the *Hindi*-translated SMMS Caesarean Births was calculated as 108.5 (Area under ROC curve 0.78, 95% CI 0.60–0.81, sensitivity 0.810, specificity 0.820). Higher scores meant better satisfaction.

## Discussion

Satisfaction is a subjective and multi-dimensional feeling [20,27,32], and lke other scales measuring satisfaction, the *Hindi-*Scale for Measuring Maternal Satisfaction too reflects it. Women who seek institutional childbirth services have a right to provide their opinion on the type of services they received, as these feedbacks become critical data to improve and make childbirth services more women-friendly and culture-friendly. However, studies show that the socio-cultural aspects of how care is delivered and how care is perceived by the recipients may affect the accuracy of measuring satisfaction [42–44]. Therefore, selection of a satisfaction questionnaire that is not only standardized, but is also close to the socio-cultural context and easily understood by the participants, is a crucial step before one starts to measure satisfaction. The SMMS Normal and Caesarean Birth scales closely reflected the Indian clinical context in terms of ward setup; the services that were covered in the questionnaire addressed all those services that are offered at an Indian public health facility. From the context of Turkish SMMS, the care responsibilities between physicians/obstetricians and nurse-midwives are similarly distributed as in India.

This study aimed to test *Hindi* translations of SMMS Normal and Caesarean Births, rigorously-tested Turkish scales, for their psychometric properties after translation, and in Indian context. The results demonstrated a 10-factor model for both *Hindi*-translated scales that resonated, for the most part, with dimensions described by the authors of the Turkish scales. However, some items in the *Hindi*-translated questionnaires were deleted based on factor-analyses, or loaded under different dimensions than the Turkish scales. This led to a slight modification in naming the 10 sub-scales. Subsequently, the *Hindi-*translated questionnaires had 36 items each. Seven items– 1, 8,9,10,11,32,33 –and six items– 1,4,15,26,35,37 –were removed from the original SMMS Normal and Caesarean Birth questionnaires, respectively; either because of very low correlation, failing to load under any factor emerging during analysis or having a correlation of 0.9 or above (measuring same concept as another item on scale). Although all factors identified in both questionnaires had fair-to-high internal reliability; in the case of SMMS Normal Births, four factors (facilities and services, postpartum care, information and involvement in decision making, and meeting the baby) had the strongest correlations with overall scores. In the case of SMMS Caesarean Births, three factors (postpartum care, meeting the baby, facilities and services) had strong correlations with final scores. However, in both questionnaires, deletion of factors with the weakest correlations did not significantly alter the overall internal reliability of the scales.

Although quality of childbirth services has been a growing concern in India and in other low- and middle-income countries [45–48], most of the literature on this comes from policy documents such as *Kalakalp* programme for improving cleanliness, hygiene and infection control in a health facility [49]; Labour Room Quality Improvement Initiative (*LAQSHYA)* [50], observational studies [51–53] or development partners’ reports [15]. Quality scientific studies where the Indian women themselves recounted their institutional childbirth experiences are few, and are mostly qualitative in nature [19,36,54]. While the qualitative studies allow an in-depth exploration of factors affecting women's satisfaction with services, having a small sample size makes it nearly impossible to generalize findings from such studies over large population, for example India. The tool has been validated with the intention to introduce a measurable feedback option for Indian women attending institutional childbirth services. The sub-scales identified in the *Hindi* SMMS have all been identified as key indicators for women's satisfaction–individually or as a group–to be associated with having satisfaction with childbirth services:

Hospital facilities such as having everything easily accessible, clear direction, safe place to stay for women and family members have been reported from studies from Low and Middle income countries (LMIC) as well as High Income Countries (HIC) [55–57]. Having opportunity to communicate with care providers is known to alleviate women's anxiety and improves their satisfaction; whereas having a bad communication experience is known to increase dissatisfaction among postnatal women [47,58,59]. Women also have expectations of being well looked after by their care providers while remaining involved and having control over their birth process. [60–62]. Having respect and privacy during labour and birth has been globally acknowledged as being very important to women [63,64]. Having support from the care providers while women make the role transition into motherhood [65,66] is also known to influence women's overall birth experience; which in turn has been closely linked to satisfaction with childbirth services [60,67]. Therefore, it can be interpreted that the *Hindi SMMS* scales capture the accepted indicators of measuring satisfaction. The dimension reduction exercise during this study eliminated seven and six items from the original Turkish tool; improving the intra-scale correlation indices. However, more research using this questionnaire is required to consolidate or challenge present study's findings. Furthermore, the clinical utility of administering this scale to assess contribution in improving quality of childbirth services is recommended.

## Strengths and limitations of the study

The strengths of this study lie in its large sample size and the rigour of the data collection methods. The demographic characteristics of the study participants resemble the general women’s population in the reproductive age group as reflected in government reports [33,68]. To the best of the authors’ knowledge, this is the first study to validate a standard childbirth satisfaction scale among *Hindi*-speaking Indian women. To the best of our knowledge, this is also the first study validating SMMS outside Turkey; and therefore draws all comparisons of our findings only with the original tool's psychometric properties. The biggest limitation of this study is the lack of establishing convergent validity against a standardised *Hindi* questionnaire measuring satisfaction. Also, the responses provided in the questionnaire are the expressed opinions of the respondents, and, as is true with all survey studies, there may be some response bias.

## Conclusion

There is a need to introduce more formal feedback mechanisms for Indian women who undergo institutional childbirths. The *Hindi-*translated questionnaires have proven to be valid and administrable to *Hindi*-speaking women; both literate and illiterate. The subscales also provide an insight on how women perceive the facilities and services, information they received and initiation of the bonding process with their newborn babies. However, further studies in *Hindi-*speaking Indian regions will be beneficial to understand the applicability and clinical usefulness of the scales.

## Supporting information

S1 Table Scale for Measuring Maternal Satisfaction-Caesarean section: Original Turkish scale translated in English. (PDF)

S2 Table Scale for Measuring Maternal Satisfaction-Normal birth: Original Turkish scale translated in English. (PDF)

S3 Table Psychometrically validated *Hindi*-translated 36-item scale for Measuring Maternal Satisfaction- Normal birth and Caesarean section. (PDF)
