# Supplementary material for: Evaluation of the psychometric properties of Hindi-translated Scale for Measuring Maternal Satisfaction among postnatal women in Chhattisgarh, India
Source: PLoS One. 2019 Jan 29;14(1):e0211364. doi: 10.1371/journal.pone.0211364 (PMC6352900; doi:10.1371/journal.pone.0211364)
Supplement: S1 Table — (PDF) [file pone.0211364.s001.pdf]

THE SCALE FOR MEASURING MATERNAL SATISFACTION IN CESAREAN BIRTH (SMMS-cesarean birth)

This questionnaire evaluates your satisfaction with the care you received during birth as well as your hospital stay after birth. Please read following statements and decide the degree of your agreement given next to each question. Then, mark the box with a cross that describes your experience best to indicate your answer in the next columns. Be careful not to leave the question blank. Your identity and your answers will be confidential. Thank you for participating.

1. The number of doctors, midwives and nurses involved in my care was enough during my hospital stay.	Strongly Disagree ☐	Partially Disagree ☐	Undecided ☐	Agree ☐	Strongly Agree ☐
2. The doctors, midwives and nurses involved in my birth treated me/behaved well.	Strongly Disagree ☐	Partially Disagree ☐	Undecided ☐	Agree ☐	Strongly Agree ☐
3. The doctors, midwives and nurses involved in my birth treated my family well.	Strongly Disagree ☐	Partially Disagree ☐	Undecided ☐	Agree ☐	Strongly Agree ☐
4. I believe that doctors have done necessary medical interventions during childbirth	Strongly Disagree ☐	Partially Disagree ☐	Undecided ☐	Agree ☐	Strongly Agree ☐
5. I was taken in the operating room for cesarean birth without delay at the scheduled time.	Strongly Disagree ☐	Partially Disagree ☐	Undecided ☐	Agree ☐	Strongly Agree ☐
6. Nurses spent enough time to prepare me for cesarean birth.	Strongly Disagree ☐	Partially Disagree ☐	Undecided ☐	Agree ☐	Strongly Agree ☐
7. The nurses spent enough time to meet my needs before cesarean birth	Strongly Disagree ☐	Partially Disagree ☐	Undecided ☐	Agree ☐	Strongly Agree ☐
8. Everyone told me just what I should do before cesarean birth.					
9. I'd like to have had more help to reduce my stress before cesarean birth.	Strongly Disagree ☐	Partially Disagree ☐	Undecided ☐	Agree ☐	Strongly Agree ☐
10. My family should have received more attention to reduce their stress before cesarean birth.	Strongly Disagree ☐	Partially Disagree ☐	Undecided ☐	Agree ☐	Strongly Agree ☐
11. I knew which doctors and midwives & nurses would be responsible from my care before cesarean birth.	Strongly Disagree ☐	Partially Disagree ☐	Undecided ☐	Agree ☐	Strongly Agree ☐
12. I was informed about all necessary procedures before cesarean birth.	Strongly Disagree ☐	Partially Disagree ☐	Undecided ☐	Agree ☐	Strongly Agree ☐
13. My partner/family was informed about all necessary procedures before cesarean birth.	Strongly Disagree ☐	Partially Disagree ☐	Undecided ☐	Agree ☐	Strongly Agree ☐
14. The doctors and midwives & nurses took into account everything I said before cesarean birth.	Strongly Disagree ☐	Partially Disagree ☐	Undecided ☐	Agree ☐	Strongly Agree ☐
15. Doctors and nurses explained me everything about cesarean birth before operation.	Strongly Disagree ☐	Partially Disagree ☐	Undecided ☐	Agree ☐	Strongly Agree ☐
16. Doctors and nurses explained my partner/family everything about cesarean birth before operation.	Strongly Disagree ☐	Partially Disagree ☐	Undecided ☐	Agree ☐	Strongly Agree ☐
17. My consent was asked before performing the procedures related with my care during birth.	Strongly Disagree ☐	Partially Disagree ☐	Undecided ☐	Agree ☐	Strongly Agree ☐
18. Consent of my partner / family was asked before performing the procedures related with my care during birth when necessary	Strongly Disagree ☐	Partially Disagree ☐	Undecided ☐	Agree ☐	Strongly Agree ☐
19. After birth, I'd like to hold my baby earlier.	Strongly Disagree ☐	Partially Disagree ☐	Undecided ☐	Agree ☐	Strongly Agree ☐
20. After birth, my family would love to be able to see the baby earlier.	Strongly Disagree ☐	Partially Disagree ☐	Undecided ☐	Agree ☐	Strongly Agree ☐
21. After birth, I'd like to breast feed my baby earlier.	Strongly Disagree ☐	Partially Disagree ☐	Undecided ☐	Agree ☐	Strongly Agree ☐

22. Some more things could have been done to reduce my pain and discomfort after birth.	Strongly Disagree ☐	Partially Disagree ☐	Undecided ☐	Agree ☐	Strongly Agree ☐
23. Nurses met my needs adequately during the days after birth.	Strongly Disagree ☐	Partially Disagree ☐	Undecided ☐	Agree ☐	Strongly Agree ☐
24. Nurses spent enough time to give information about my own care after birth.	Strongly Disagree ☐	Partially Disagree ☐	Undecided ☐	Agree ☐	Strongly Agree ☐
25. Nurses spent enough time to give information about the care of my baby.	Strongly Disagree ☐	Partially Disagree ☐	Undecided ☐	Agree ☐	Strongly Agree ☐
26. Nurses spent enough time to help breastfeeding.	Strongly Disagree ☐	Partially Disagree ☐	Undecided ☐	Agree ☐	Strongly Agree ☐
27. The information received from different caregivers about self-care and baby care was consistent.	Strongly Disagree ☐	Partially Disagree ☐	Undecided ☐	Agree ☐	Strongly Agree ☐
28. The room in which I stayed during preparation for cesarean was clean and adequate to meet my needs.	Strongly Disagree ☐	Partially Disagree ☐	Undecided ☐	Agree ☐	Strongly Agree ☐
29. The room in which I stayed after birth was comfortable and adequate to meet my needs.	Strongly Disagree ☐	Partially Disagree ☐	Undecided ☐	Agree ☐	Strongly Agree ☐
30. The room in which I stayed after birth was suitable for the visits of my family and friends.	Strongly Disagree ☐	Partially Disagree ☐	Undecided ☐	Agree ☐	Strongly Agree ☐
31. My family had a proper and comfortable place in the hospital to rest and wait during birth.	Strongly Disagree ☐	Partially Disagree ☐	Undecided ☐	Agree ☐	Strongly Agree ☐
32. We could easily find everything we needed in hospital.	Strongly Disagree ☐	Partially Disagree ☐	Undecided ☐	Agree ☐	Strongly Agree ☐
33. The food service was good at hospital.	Strongly Disagree ☐	Partially Disagree ☐	Undecided ☐	Agree ☐	Strongly Agree ☐
34. There were people coming in and out of my room unnecessarily during preparation for cesarean.	Strongly Disagree ☐	Partially Disagree ☐	Undecided ☐	Agree ☐	Strongly Agree ☐
35. There were people coming in and out of my room unnecessarily after birth.	Strongly Disagree ☐	Partially Disagree ☐	Undecided ☐	Agree ☐	Strongly Agree ☐
36. Health-care personnel showed respect to my privacy during their practices.	Strongly Disagree ☐	Partially Disagree ☐	Undecided ☐	Agree ☐	Strongly Agree ☐
37. Special moments I lived with my family before and after cesarean birth were interrupted by medical staff because of routine interventions that could be delayed easily.	Strongly Disagree ☐	Partially Disagree ☐	Undecided ☐	Agree ☐	Strongly Agree ☐
38. I could not get any better care in this hospital.	Strongly Disagree ☐	Partially Disagree ☐	Undecided ☐	Agree ☐	Strongly Agree ☐
39. My birth experience was completely as I had expected and hoped.	Strongly Disagree ☐	Partially Disagree ☐	Undecided ☐	Agree ☐	Strongly Agree ☐
40. The cesarean birth took longer than I had expected.	Strongly Disagree ☐	Partially Disagree ☐	Undecided ☐	Agree ☐	Strongly Agree ☐
41. I had not expected to have some of the medical interventions used at my birth.	Strongly Disagree ☐	Partially Disagree ☐	Undecided ☐	Agree ☐	Strongly Agree ☐
42. This birth was one of the most beautiful experiences in my life.	Strongly Disagree ☐	Partially Disagree ☐	Undecided ☐	Agree ☐	Strongly Agree ☐

(Gungor & Beji 2012)

SCORING AND EVALUATION

- The Scale for Measuring Maternal Satisfaction in Cesarean Birth (SMMS- cesarean birth) consists of 42 items and 10 subscales. Item numbers for each subscale are given in the table below.
- Responses are coded as in the following.
  1-Strongly Disagree, **2-**Partially Disagree, **3**-Undecided, **4**- Agree, **5**- Strongly Agree
- The scale has 12 negatively worded items (8,9,10,19,20,21,22,34,35,37,40,41) so that these items should be recoded before calculation of scores as in the following.
  5-Strongly Disagree, **4-**Partially Disagree, **3**-Undecided, **2**- Agree, **1**- Strongly Agree
- Subject scores on the total scale can range from 42 to 210 points.
- The cut-off score was calculated as 146.5 for the SMMS-cesarean birth and scores above the cut-off value indicate greater satisfaction.
- The original language of the scale is Turkish. Therefore psychometric properties of the English version of SMSS should be tested.

"The Scale for Measuring Maternal Satisfaction in Cesarean Birth"		Item No
Factor 1	Perception of Health Professionals	1,2,3,4,5
Factor 2	Preparation for Cesarean	6,7
Factor 3	Comforting	8,9,10
Factor 4	Information and Involvement in Decision Making	11,12,13,14,15,16,17,18
Factor 5	Meeting Baby	19,20,21
Factor 6	Postpartum Care	22,23,24,25,26,27
Factor 7	Hospital Room	28,29,30
Factor 8	Hospital Facilities	31,32,33
Factor 9	Respect for Privacy	34,35,36,37
Factor 10	Meeting Expectations	38,39,40,41,42

<u>Reference:</u>

Gungor, I., Beji, N.K., Development and psychometric testing of the scales for measuring maternal satisfaction in normal and caesarean birth. Midwifery, May 2012, 28(3):348-357.

<u>Contact:</u>

Assist. Prof. İlkay GÜNGÖR
Istanbul University Florence Nightingale Faculty of Nursing
Department of Women Health and Diseases Nursing
Abide-i Hurriyet cad. 34381, Sisli, Istanbul, Turkey
Phone: (+90) 4400000/ 27088 Fax: (+90) 2122244990
e-mail: ilkay1979@yahoo.com
