# Supplementary material for: Evaluation of the psychometric properties of Hindi-translated Scale for Measuring Maternal Satisfaction among postnatal women in Chhattisgarh, India
Source: PLoS One. 2019 Jan 29;14(1):e0211364. doi: 10.1371/journal.pone.0211364 (PMC6352900; doi:10.1371/journal.pone.0211364)
Supplement: S3 Table — (PDF) [file pone.0211364.s003.pdf]

सामान्य शिशु जन्म के प्रति माता की संतुष्टि मापने का पैमाना (SMMS सामान्य शिशु जन्म)

निर्देश:
यह प्रश्नावली प्रसव तथा शिशु जन्म के समय, तथा हस्पताल में आप के ठहरने के समय आप को मिली देखभाल के प्रति आप की संतुष्टि का मूल्यांकन करने के लिए है. कृपया नीचे दिए हुए वक्तव्यों को पढ़ें तथा आप की सहमति की हद को प्रत्येक प्रश्न के नीचे दिए जवाबों के अनुसार निर्धारित करें. इसके बाद, अपना उत्तर उस अंक के चारों ओर एक घेरा बनाकर दीजिये जो आपके अनुभव को सबसे अच्छी तरह परिभाषित करता हो. कृपया ध्यान रखें कि कोई भी प्रश्न बिना उत्तर के नहीं छूट जाये. आश्वस्त रहें कि आप की पहचान तथा आपके उत्तर गुप्त रखे जाएंगे। सर्वेक्षण में भाग लेने के लिए आपका धन्यवाद!!

ND01.	मेरे प्रसव के समय मेरी देखभाल में लगे सब डॉक्टर, नर्सें और मिडवाइफ़ें मेरे साथ सम्मान से पेश आये/ मुझसे अच्छा सलूक किये<br>1. पूर्णतया असहमत 2. थोड़ा असहमत 3. अनिश्चित 4. सहमत 5. पूर्णतया सहमत
ND02.	मेरे प्रसव के समय मेरी देखभाल में लगे सब डॉक्टर, नर्सें और मिडवाइफ़ें मेरे परिवार वालों से सम्मान से पेश आये<br>1. पूर्णतया असहमत 2. थोड़ा असहमत 3. अनिश्चित 4. सहमत 5. पूर्णतया सहमत
ND03.	मेरा मानना है कि मेरे प्रसव के दौरान डॉक्टरों ने सभी संभव आवश्यक चिकित्सकीय हस्तक्षेप किये<br>1. पूर्णतया असहमत 2. थोड़ा असहमत 3. अनिश्चित 4. सहमत 5. पूर्णतया सहमत
ND04.	नर्सेज तथा मिडवाइफ्स ने मुझे प्रसव-पीड़ा सहने में मदद करने के लिए पर्याप्त समय (जितना मुझे चाहिए था) व्यतीत किया<br>1. पूर्णतया असहमत 2. थोड़ा असहमत 3. अनिश्चित 4. सहमत 5. पूर्णतया सहमत
ND05.	नर्सों ने प्रसव तथा शिशु जन्म के समय मेरी आवश्यकताओं को पूरा करने हेतु पर्याप्त समय (जितना मुझे चाहिए था) दिया<br>1. पूर्णतया असहमत 2. थोड़ा असहमत 3. अनिश्चित 4. सहमत 5. पूर्णतया सहमत
ND06.	हर किसी ने मुझे बताया कि शिशु जन्म के समय मुझे यथार्थतः/ दरअसल क्या करना है<br>1. पूर्णतया असहमत 2. थोड़ा असहमत 3. अनिश्चित 4. सहमत 5. पूर्णतया सहमत
ND07.	प्रसव दौरान मेरा दर्द कम करने के लिए कुछ और भी किया जा सकता था (मसाज, ध्यान/ meditation इत्यादि)<br>1. पूर्णतया असहमत 2. थोड़ा असहमत 3. अनिश्चित 4. सहमत 5. पूर्णतया सहमत
ND08.	मेरे पति/ परिवार ने मेरे प्रसव तथा शिशु जन्म के समय होने वाली सभी प्रक्रियाओं (प्रोसीजर्स) के बारे में जानकारी (आसानी से) हासिल की<br>1. पूर्णतया असहमत 2. थोड़ा असहमत 3. अनिश्चित 4. सहमत 5. पूर्णतया सहमत
ND09.	डॉक्टरों, मिडवाइफ़ों और नर्सों ने शिशु जन्म दौरान मेरी कही हुई हर बात का ध्यान रखा<br>1. पूर्णतया असहमत 2. थोड़ा असहमत 3. अनिश्चित 4. सहमत 5. पूर्णतया सहमत
ND10.	मैं जानती थी कि कौन से डॉक्टर, नर्सें तथा मिडवाइफ़ें शिशु जन्म समय मेरी देखभाल के जिम्मेदार होंगे<br>1. पूर्णतया असहमत 2. थोड़ा असहमत 3. अनिश्चित 4. सहमत 5. पूर्णतया सहमत
ND11.	डॉक्टरों और नर्सों ने मुझे मेरे शिशु के जन्म सम्बन्धी हर नयी स्थिति के बारे में समझाया<br>1. पूर्णतया असहमत 2. थोड़ा असहमत 3. अनिश्चित 4. सहमत 5. पूर्णतया सहमत
ND12	डॉक्टरों और नर्सों ने मेरे परिवार वालों को मेरे शिशु के जन्म सम्बन्धी हर नयी स्थिति के बारे में समझाया<br>1. पूर्णतया असहमत 2. थोड़ा असहमत 3. अनिश्चित 4. सहमत 5. पूर्णतया सहमत
ND13	शिशु जन्म के समय मेरी देखभाल सम्बन्धी प्रक्रियाएं (प्रोसीजर्स) करने से पहले मेरी अनुमति ली गयी<br>1. पूर्णतया असहमत 2. थोड़ा असहमत 3. अनिश्चित 4. सहमत 5. पूर्णतया सहमत
ND14	शिशु जन्म के समय (जब आवश्यक था, तब) मेरी देखभाल सम्बन्धी प्रक्रियाएं (प्रोसीजर्स) करनेसे पहले मेरे पति/ परिवार वालों की अनुमति ली गयी<br>1. पूर्णतया असहमत 2. थोड़ा असहमत 3. अनिश्चित 4. सहमत 5. पूर्णतया सहमत

ND15	शिशु जन्म पश्चात, मैं अपने बच्चे को और भी जल्दी अपनी गोद में लेना चाहती थी<br>1. पूर्णतया असहमत 2. थोड़ा असहमत 3. अनिश्चित 4. सहमत 5. पूर्णतया सहमत
ND16	शिशु जन्म पश्चात, मेरा परिवार मेरे बच्चे को और भी जल्दी देखना पसंद करता<br>1. पूर्णतया असहमत 2. थोड़ा असहमत 3. अनिश्चित 4. सहमत 5. पूर्णतया सहमत
ND17	शिशु जन्म पश्चात, मुझे अच्छा लगता अगर मैं अपने बच्चे को और भी जल्दी स्तनपान करा पाती<br>1. पूर्णतया असहमत 2. थोड़ा असहमत 3. अनिश्चित 4. सहमत 5. पूर्णतया सहमत
ND18	शिशु जन्म के बाद के दिनों में नर्सों ने मेरी ज़रूरतों का पर्याप्त (जितना मुझे चाहिए था) ध्यान रखा<br>1. पूर्णतया असहमत 2. थोड़ा असहमत 3. अनिश्चित 4. सहमत 5. पूर्णतया सहमत
ND19	शिशु जन्म के बाद स्वयं की देखभाल के बारे में मुझे जानकारी देने के लिए नर्सों ने पर्याप्त (जितना मुझे चाहिए था) समय दिया<br>1. पूर्णतया असहमत 2. थोड़ा असहमत 3. अनिश्चित 4. सहमत 5. पूर्णतया सहमत
ND20	शिशु जन्म के बाद मेरे शिशु की देखभाल के विषय में जानकारी देने के लिए नर्सों ने पर्याप्त समय (जितना मुझे चाहिए था) दिया<br>1. पूर्णतया असहमत 2. थोड़ा असहमत 3. अनिश्चित 4. सहमत 5. पूर्णतया सहमत
ND21	स्तनपान करवाने में मेरी मदद करने के लिए नर्सों ने पर्याप्त समय (जितना मुझे चाहिए था) दिया<br>1. पूर्णतया असहमत 2. थोड़ा असहमत 3. अनिश्चित 4. सहमत 5. पूर्णतया सहमत
ND22	अलग अलग स्वास्थ्यकर्मियों (डॉक्टरों, नर्सों, मिडवाइफ़ों) से स्वयं की और शिशु की देखभाल से सम्बंधित एक ही जैसी जानकारी मिली<br>1. पूर्णतया असहमत 2. थोड़ा असहमत 3. अनिश्चित 4. सहमत 5. पूर्णतया सहमत
ND23	प्रसव के दौरान मैं जिस कमरे में रुकी, वो साफ़ सुथरा और मेरी ज़रूरतों के हिसाब से पर्याप्त था<br>1. पूर्णतया असहमत 2. थोड़ा असहमत 3. अनिश्चित 4. सहमत 5. पूर्णतया सहमत
ND24	वो कमरा जहां मैंने अपने शिशु को जन्म दिया,साफ़ सुथरा और आराम देह था<br>1. पूर्णतया असहमत 2. थोड़ा असहमत 3. अनिश्चित 4. सहमत 5. पूर्णतया सहमत
ND25	शिशु जन्म पश्चात मैं जिस कमरे में रुकी, वो आरामदेह और मेरी ज़रूरतों के हिसाब से पर्याप्त था<br>1. पूर्णतया असहमत 2. थोड़ा असहमत 3. अनिश्चित 4. सहमत 5. पूर्णतया सहमत
ND26	शिशु जन्म पश्चात मैं जिस कमरे में रुकी वो इस लायक था कि मेरा परिवार और मित्र मुझसे मिलने आ सकें<br>1. पूर्णतया असहमत 2. थोड़ा असहमत 3. अनिश्चित 4. सहमत 5. पूर्णतया सहमत
ND27	हॉस्पिटल में दी गयी भोजन-सुविधा अच्छी थी<br>1. पूर्णतया असहमत 2. थोड़ा असहमत 3. अनिश्चित 4. सहमत 5. पूर्णतया सहमत
ND28	प्रसव दौरान कुछ लोग अनावश्यक ही मेरे कमरे में आ जा रहे थे<br>1. पूर्णतया असहमत 2. थोड़ा असहमत 3. अनिश्चित 4. सहमत 5. पूर्णतया सहमत
ND29	शिशु जन्म पश्चात कुछ लोग अनावश्यक ही मेरे कमरे में आ जा रहे थे<br>1. पूर्णतया असहमत 2. थोड़ा असहमत 3. अनिश्चित 4. सहमत 5. पूर्णतया सहमत
ND30	अपना कार्य करते समय चिकित्सा-कर्मियों ने मेरी एकान्तता (प्राइवेसी) बनाये रखने के प्रति सम्मान दिखाया<br>1. पूर्णतया असहमत 2. थोड़ा असहमत 3. अनिश्चित 4. सहमत 5. पूर्णतया सहमत
ND31	शिशु जन्म के दौरान और बादमें जो ख़ास पल मैंने अपने परिवार वालों के साथ बिताये वो चिकित्सा-कर्मियों द्वारा ऐसी प्रक्रियाएं (प्रोसीजर) करने के लिए अवरुद्ध किये गए जो आसानी से बाद में भी की जा सकती थीं<br>1. पूर्णतया असहमत 2. थोड़ा असहमत 3. अनिश्चित 4. सहमत 5. पूर्णतया सहमत
ND32	इस हॉस्पिटल में मुझे इससे अच्छी देखभाल नहीं मिल सकती थी<br>1. पूर्णतया असहमत 2. थोड़ा असहमत 3. अनिश्चित 4. सहमत 5. पूर्णतया सहमत

ND33	मेरा शिशु जन्मका अनुभव बिलकुल वैसा ही था जैसे की मुझे आशा और अपेक्षा थी<br>1. पूर्णतया असहमत 2. थोड़ा असहमत 3. अनिश्चित 4. सहमत 5. पूर्णतया सहमत
ND34	प्रसव मेरी अपेक्षा से ज़्यादा लंबा था<br>1. पूर्णतया असहमत 2. थोड़ा असहमत 3. अनिश्चित 4. सहमत 5. पूर्णतया सहमत
ND35	मेरे प्रसव के दौरान किये गए कुछ चिकित्सकीय हस्तक्षेपों की मैंने अपेक्षा नहीं की थी<br>1. पूर्णतया असहमत 2. थोड़ा असहमत 3. अनिश्चित 4. सहमत 5. पूर्णतया सहमत
ND36	यह शिशु-जन्म मेरे जीवन के सबसे सुन्दर अनुभवों में से एक था<br>1. पूर्णतया असहमत 2. थोड़ा असहमत 3. अनिश्चित 4. सहमत 5. पूर्णतया सहमत

सीज़ेरियन द्वारा शिशु जन्म के प्रति माता की संतुष्टि मापने का पैमाना (SMMS सीज़ेरियन शिशु जन्म)	
**निर्देश:**<br>यह प्रश्नावली प्रसव तथा शिशु जन्म के समय, तथा हस्पताल में आप के ठहरने के समय आप को मिली देखभाल के प्रति आप की संतुष्टि का मूल्यांकन करने के लिए है. कृपया नीचे दिए हुए वक्तव्यों को पढ़ें तथा आप की सहमति की हद को प्रत्येक प्रश्न के नीचे दिए जवाबों के अनुसार निर्धारित करें. इसके बाद, अपना उत्तर उस अंक के चारों ओर एक घेरा बनाकर दीजिये जो आपके अनुभव को सबसे अच्छी तरह परिभाषित करता हो. कृपया ध्यान रखें कि कोई भी प्रश्न बिना उत्तर के नहीं छूट जाये. आश्वस्त रहें कि आप की पहचान तथा आपके उत्तर गुप्त रखे जाएंगे। सर्वेक्षण में भाग लेने के लिए आपका धन्यवाद!!	
CS01.	मेरे प्रसव के समय मेरी देखभाल में लगे सब डॉक्टर, नर्सें और मिडवाइफ़ें मेरे साथ सम्मान से पेश आये/ मुझसे अच्छा सलूक किये<br>1. पूर्णतया असहमत 2. थोड़ा असहमत 3. अनिश्चित 4. सहमत 5. पूर्णतया सहमत
CS02.	मेरे प्रसव के समय मेरी देखभाल में लगे सब डॉक्टर, नर्सें और मिडवाइफ़ें मेरे परिवार वालों से सम्मान से पेश आये<br>1. पूर्णतया असहमत 2. थोड़ा असहमत 3. अनिश्चित 4. सहमत 5. पूर्णतया सहमत
CS03.	मुझे निर्धारित समय पर, बिना किसी देर के, सीज़ेरियन जन्म हेतु ऑपरेशन कक्ष के भीतर ले जाया गया<br>1. पूर्णतया असहमत 2. थोड़ा असहमत 3. अनिश्चित 4. सहमत 5. पूर्णतया सहमत
CS04.	नर्सों ने मुझे सीज़ेरियन जन्म हेतु (शारीरिक रूप से) तैयार करने के लिए पर्याप्त समय बिताया<br>1. पूर्णतया असहमत 2. थोड़ा असहमत 3. अनिश्चित 4. सहमत 5. पूर्णतया सहमत
CS05.	सीज़ेरियन जन्म से पहले नर्सों ने मेरी आवश्यकताओं को पूरा करने के लिए पर्याप्त समय बिताया<br>1. पूर्णतया असहमत 2. थोड़ा असहमत 3. अनिश्चित 4. सहमत 5. पूर्णतया सहमत
CS06.	हर किसी ने मुझे बताया कि सीजेरियन जन्म से पहले मुझे यथार्थतः/ दरअसल क्या करना है<br>1. पूर्णतया असहमत 2. थोड़ा असहमत 3. अनिश्चित 4. सहमत 5. पूर्णतया सहमत
CS07.	मुझे अच्छा लगता अगर सीजेरियन जन्म से पहले मेरा तनाव कम करने के लिए मुझे और मदद दी जाती<br>1. पूर्णतया असहमत 2. थोड़ा असहमत 3. अनिश्चित 4. सहमत 5. पूर्णतया सहमत
CS08.	सीजेरियन जन्म से पहले मेरे परिवार वालों का तनाव कम करने के ऊपर और ध्यान दिया जाना चाहिए था<br>1. पूर्णतया असहमत 2. थोड़ा असहमत 3. अनिश्चित 4. सहमत 5. पूर्णतया सहमत
CS09.	मैं जानती थी कि कौन से डॉक्टर, नर्सें तथा मिडवाइफ़ें सीजेरियन जन्म से पहले मेरी देखभाल के जिम्मेदार होंगे<br>1. पूर्णतया असहमत 2. थोड़ा असहमत 3. अनिश्चित 4. सहमत 5. पूर्णतया सहमत
CS10.	मेरे पति/ परिवार वालों को सीजेरियन जन्म से पहले होने वाली सभी आवश्यक प्रक्रियाओं के बारे में जानकारी दी गयी<br>1. पूर्णतया असहमत 2. थोड़ा असहमत 3. अनिश्चित 4. सहमत 5. पूर्णतया सहमत
CS11.	डॉक्टरों, मिडवाइफ़ों और नर्सों ने सीजेरियन जन्म के पहले मेरी कही हुई हर बात का ध्यान रखा<br>1. पूर्णतया असहमत 2. थोड़ा असहमत 3. अनिश्चित 4. सहमत 5. पूर्णतया सहमत
CS12.	मेरे हिसाब से, डॉक्टरों और नर्सों ने मुझे ऑपरेशन से पहले सीजेरियन जन्म के बारे में सब कुछ समझा दिया था<br>1. पूर्णतया असहमत 2. थोड़ा असहमत 3. अनिश्चित 4. सहमत 5. पूर्णतया सहमत
CS13.	मेरे हिसाब से, डॉक्टरों और नर्सों ने मेरे पति/ परिवार वालों को ऑपरेशन से पहले सीजेरियन जन्म के बारे में सब कुछ समझा दिया था<br>1. पूर्णतया असहमत 2. थोड़ा असहमत 3. अनिश्चित 4. सहमत 5. पूर्णतया सहमत
CS14.	शिशु जन्म के समय मेरी देखभाल सम्बन्धी प्रक्रियाएं (प्रोसीजर्स) करने से पहले मेरी अनुमति लीगयी<br>1. पूर्णतया असहमत 2. थोड़ा असहमत 3. अनिश्चित 4. सहमत 5. पूर्णतया सहमत

CS15.	शिशु जन्म के समय (जब आवश्यक था, तब) मेरी देखभाल सम्बन्धी प्रक्रियाएं (प्रोसीजर्स) करने से पहले मेरे पति/ परिवार वालों की अनुमति ली गयी<br>1. पूर्णतया असहमत 2. थोड़ा असहमत 3. अनिश्चित 4. सहमत 5. पूर्णतया सहमत
CS16.	शिशु जन्म पश्चात, मैं अपने बच्चे को और भी जल्दी अपनी गोद में लेना चाहती थी<br>1. पूर्णतया असहमत 2. थोड़ा असहमत 3. अनिश्चित 4. सहमत 5. पूर्णतया सहमत
CS17.	शिशु जन्म पश्चात, मेरा परिवार मेरे बच्चे को और भी जल्दी देखना पसंद करता<br>1. पूर्णतया असहमत 2. थोड़ा असहमत 3. अनिश्चित 4. सहमत 5. पूर्णतया सहमत
CS18.	शिशु जन्म पश्चात, मुझे अच्छा लगता अगर मैं अपने बच्चे को और भी जल्दी स्तनपान करा पाती<br>1. पूर्णतया असहमत 2. थोड़ा असहमत 3. अनिश्चित 4. सहमत 5. पूर्णतया सहमत
CS19.	शिशु जन्म पश्चात मेरा दर्द और असुविधा कम करने के लिए और भी कुछ किया जा सकता था<br>1. पूर्णतया असहमत 2. थोड़ा असहमत 3. अनिश्चित 4. सहमत 5. पूर्णतया सहमत
CS20.	शिशु जन्म के बाद के दिनों में नर्सोंने मेरी ज़रूरतों का पर्याप्त (जितना मुझे चाहिए था) ध्यान रखा<br>1. पूर्णतया असहमत 2. थोड़ा असहमत 3. अनिश्चित 4. सहमत 5. पूर्णतया सहमत
CS21.	शिशु जन्म के बाद स्वयं की देखभाल के बारे में मुझे जानकारी देने के लिए नर्सों ने पर्याप्त (जितना मुझे चाहिए था) समय दिया<br>1. पूर्णतया असहमत 2. थोड़ा असहमत 3. अनिश्चित 4. सहमत 5. पूर्णतया सहमत
CS22.	मेरे शिशु की देखभाल के विषय में जानकारी देने के लिए नर्सों ने पर्याप्त (जितना मुझे चाहिए था) समय दिया<br>1. पूर्णतया असहमत 2. थोड़ा असहमत 3. अनिश्चित 4. सहमत 5. पूर्णतया सहमत
CS23.	अलग अलग ध्यान रखने वालों (डॉक्टरों, नर्सों, मिडवाइफ़ों) से स्वयं की और शिशु की देखभाल से सम्बंधित एक ही जैसी जानकारी मिली<br>1. पूर्णतया असहमत 2. थोड़ा असहमत 3. अनिश्चित 4. सहमत 5. पूर्णतया सहमत
CS24.	सीजेरियन के लिए तैयारी से पहले मैं जिस कमरे में रुकी वो साफ़ सुथरा और मेरी ज़रूरतों के हिसाब से पर्याप्त (जितना मुझे चाहिए था) था<br>1. पूर्णतया असहमत 2. थोड़ा असहमत 3. अनिश्चित 4. सहमत 5. पूर्णतया सहमत
CS25.	शिशु जन्म पश्चात मैं जिस कमरे में रुकी, वो आरामदेह और मेरी ज़रूरतों के हिसाब से पर्याप्त (जितना मुझे चाहिए था) था<br>1. पूर्णतया असहमत 2. थोड़ा असहमत 3. अनिश्चित 4. सहमत 5. पूर्णतया सहमत
CS26.	शिशु जन्म पश्चात मैं जिस कमरे में रुकी वो इस लायक था कि मेरा परिवार और मित्र मुझसे मिलने आ सकें<br>1. पूर्णतया असहमत 2. थोड़ा असहमत 3. अनिश्चित 4. सहमत 5. पूर्णतया सहमत
CS27.	शिशु जन्म के समय हॉस्पिटल में आराम और इंतज़ार करने के लिए एक आरामदेह और ठीक ठाक स्थान मेरे परिवार को उपलब्ध कराया गया<br>1. पूर्णतया असहमत 2. थोड़ा असहमत 3. अनिश्चित 4. सहमत 5. पूर्णतया सहमत
CS28.	हॉस्पिटल में हमें जिस भी चीज़ की आवश्यकता पड़ी, वोह हम आसानीसे ढूंढ पाये<br>1. पूर्णतया असहमत 2. थोड़ा असहमत 3. अनिश्चित 4. सहमत 5. पूर्णतया सहमत
CS29.	हॉस्पिटल में दी गयी भोजन-सुविधा अच्छी थी<br>1. पूर्णतया असहमत 2. थोड़ा असहमत 3. अनिश्चित 4. सहमत 5. पूर्णतया सहमत
CS30.	प्रसव दौरान कुछ लोग अनावश्यक ही मेरे कमरे में आ जा रहे थे<br>1. पूर्णतया असहमत 2. थोड़ा असहमत 3. अनिश्चित 4. सहमत 5. पूर्णतया सहमत

CS31.	अपना कार्य करते समय चिकित्सा-कर्मियों ने मेरी एकान्तता (प्राइवेसी) बनाये रखने के प्रति सम्मान दिखाया<br>1. पूर्णतया असहमत 2. थोड़ा असहमत 3. अनिश्चित 4. सहमत 5. पूर्णतया सहमत
CS32.	इस हॉस्पिटल में मुझे इससे अच्छी देखभाल नहीं मिल सकती थी<br>1. पूर्णतया असहमत 2. थोड़ा असहमत 3. अनिश्चित 4. सहमत 5. पूर्णतया सहमत
CS33.	मेरा शिशु जन्म का अनुभव बिलकुल वैसा ही था जैसे की मुझे आशा और अपेक्षा थी<br>1. पूर्णतया असहमत 2. थोड़ा असहमत 3. अनिश्चित 4. सहमत 5. पूर्णतया सहमत
CS34.	प्रसव मेरी अपेक्षा से ज़्यादा लंबा था<br>1. पूर्णतया असहमत 2. थोड़ा असहमत 3. अनिश्चित 4. सहमत 5. पूर्णतया सहमत
CS35.	मेरे प्रसवके दौरान किये गए कुछ चिकित्सकीय हस्तक्षेपों की मैंने अपेक्षा नहीं की थी<br>1. पूर्णतया असहमत 2. थोड़ा असहमत 3. अनिश्चित 4. सहमत 5. पूर्णतया सहमत
CS36.	यह शिशु-जन्म मेरे जीवन के सबसे सुन्दर अनुभवों में से एक था<br>1. पूर्णतया असहमत 2. थोड़ा असहमत 3. अनिश्चित 4. सहमत 5. पूर्णतया सहमत
